# Complications of Diabetes

**DOI:** 10.1155/2015/189525

**Published:** 2015-07-12

**Authors:** Konstantinos Papatheodorou, Maciej Banach, Michael Edmonds, Nikolaos Papanas, Dimitrios Papazoglou

**Affiliations:** ^1^Diabetes Clinic, Second Department of Internal Medicine, Democritus University of Thrace, 68100 Alexandroupolis, Greece; ^2^Department of Hypertension, Chair of Nephrology and Hypertension, Medical University of Lodz, 90-419 Lodz, Poland; ^3^Diabetic Foot Clinic, King's College Hospital, London SE59RS, UK

Diabetes is justly recognized as an emerging global epidemic, representing one of the leading causes of morbidity and mortality worldwide. Hyperglycemia, the common characteristic of both type 1 diabetes mellitus (T1DM) and type 2 diabetes mellitus (T2DM), has the potential to cause serious complications due to its insidious and chronic nature. The present special issue has been designed to publish original and review articles highlighting recent fundamental advances in our understanding of diabetic complications. Emphasis has been given on the underlying molecular mechanisms, the new technologies that have been introduced to facilitate early diagnosis, and the new potential therapies for these complications. There are 30 articles in total, which cover 6 thematic areas: pathogenesis of diabetic complications, diabetic neuropathy, nephropathy, retinopathy, macrovascular complications, and miscellaneous complications.

*(a) Pathogenesis of Diabetic Complications*. There is growing evidence that the underlying mechanisms in the pathogenesis of diabetic complications include oxidative stress created by the overproduction of reactive oxygen species (ROS) and defects in the insulin signal transduction pathway in which ceramide, a bioactive sphingolipid, may have an important inhibitory effect [1, 2].

In a paper of this special issue entitled “Pathogenesis of Chronic Hyperglycemia: From Reductive Stress to Oxidative Stress,” L.-J. Yan reviews the role of reductive stress in the generation of oxidative stress. This review provides an interesting and novel approach on the pathogenesis of diabetic complications, emphasizing the importance of reductive stress in this complex process.

Another article included in this special issue by M. Mihailović et al. entitled “Protective Effects of the Mushroom* Lactarius deterrimus* Extract on Systemic Oxidative Stress and Pancreatic Islets in Streptozotocin-Induced Diabetic Rats” describes a study designed to assess the effects of mushroom* Lactarius deterrimus* extract on pancreatic *β*-cells and on systemic oxidative stress. This study showed that diabetic rats treated with the abovementioned extract exhibited better glycemic profile and a remarkable increase in *β*-cell mass.

In the same spirit, J.-F. Hu et al. in their paper titled “Ethanol at Low Concentration Attenuates Diabetes Induced Lung Injury in Rats Model” provide some evidence that ethanol at low concentration may prevent hyperglycemia-induced oxidative stress injury of lungs in diabetic rats. However, these results have been derived from rats, and, therefore, new studies should be designed in humans to explore such potential favorable effects.

The final paper of this thematic area is of M. E. Hansen et al. entitled “Insulin Increases Ceramide Synthesis in Skeletal Muscle.” This study highlights the key role of insulin on ceramide metabolism in skeletal muscle and suggests that there might be an insulin-induced augmentation in the expression of genes controlling ceramide biosynthesis. Obviously, these results are promising and may help towards defining a novel therapeutic target. 

*(b) Diabetic Neuropathy*. One of the most common complications of diabetes is diabetic peripheral neuropathy (DPN). Moreover, not only can chronic hyperglycemia have various negative effects on the central nervous system, but it can also cause gastroparesis [3, 4]. The role of glycemic variability (GV), which is recognized as an important component of the overall glycemic control, in diabetic neuropathy is also under investigation [5].

The paper entitled “Relationship between Autonomic Nervous System Function and Continuous Interstitial Glucose Measurement in Patients with Type 2 Diabetes” by S. Kalopita et al. aimed to examine if there is an association between the function of ANS and GV in patients with T2DM. This study showed that heart rate variability (HRV) was inversely associated with GV. Further prospective studies are required to ascertain the role of GV in the pathogenesis of ANS dysfunction.

In their paper titled “Decreased Neuronal Bursting and Phase Synchrony in the Hippocampus of Streptozotocin Diabetic Rats,” Z. Qiao et al. explored the effects of an active fragment of amyloid precursor protein (APP 17-mer peptide) on the function of the hippocampus in streptozotocin-diabetic rats. Authors found that treatment with APP 17-mer peptide partially reversed the effects of diabetes on parameters of burst activity. These findings provide a more complete picture on the effects of diabetes on the function of hippocampus and encourage further enquiry into the changes of neuronal function in diabetes.

The study entitled “The Influence of Peripheral Neuropathy, Gender, and Obesity on the Postural Stability of Patients with Type 2 Diabetes Mellitus” by A. Herrera-Rangel et al. evaluated postural stability in patients with T2DM. The authors demonstrated that measures of postural stability were positively associated with the presence of peripheral neuropathy. These results shed light on the role of diabetes in the development of balance disorders.

Turning their attention to biomarkers of early nerve damage, A. Sun et al. in their paper titled “Urinary Methylmalonic Acid as an Indicator of Early Vitamin B_12_ Deficiency and Its Role in Polyneuropathy in Type 2 Diabetes” examined the levels of urinary methylmalonic acid (uMMA) in Chinese patients with T2DM and its relationship with serum vitamin B_12_ and polyneuropathy. The authors claimed that uMMA could be used as a sensitive marker of early polyneuropathy in patients with diabetes. However, the underlying mechanism of this relationship is unknown and needs to be further addressed.

The paper entitled “Vomiting and Dysphagia Predict Delayed Gastric Emptying in Diabetic and Nondiabetic Subjects” by D. Boltin et al. investigated the predictors of delayed gastric emptying in patients with and without diabetes. This study provides a more complete picture on gastric emptying procedure and how it is affected by diabetes.

E. Søfteland et al. in their paper titled “Rectal Sensitivity in Diabetes Patients with Symptoms of Gastroparesis” documented that patients with diabetes and gastroparesis exhibited reduced rectal sensitivity and impaired heart rate variability, as compared with healthy volunteers. As the authors suggest, the evaluation of heart rate variability could be developed into a rational screening test for diabetic gastroparesis. This is a promising hypothesis that needs to be confirmed by further studies.

X. Wang et al. in their article entitled “Early Detection of Atrophy of Foot Muscles in Chinese Patients of Type 2 Diabetes Mellitus by High-Frequency Ultrasonography” examined the efficacy of ultrasonography in detecting foot muscle atrophy in Chinese T2DM patients. The study showed that indices of muscular atrophy were significantly lower in patients with DPN. The clinical implication of this study is that high-frequency ultrasonography could be used to increase the timely detection of foot muscle atrophy in T2DM patients even before DPN becomes manifest.

S. L. Misra et al. in the paper of this thematic area entitled “Peripheral Neuropathy and Tear Film Dysfunction in Type 1 Diabetes Mellitus” explored the relationship between peripheral neuropathy and the quality of ocular surface in patients with T1DM. The findings suggest that ocular surface abnormalities develop in parallel with DPN and the two conditions are strongly connected to each other. Clearly, there is a lot to learn about this issue in the future. 

*(c) Diabetic Nephropathy*. Diabetic nephropathy (DN) is the major cause of end-stage renal disease, although its pathogenesis is not fully understood. Emerging evidence suggests that epigenetic modifications and some microRNAs may play a role in the pathogenesis of DN by altering the expression of several genes and controlling certain intracellular pathways [6, 7].

The paper entitled “The Role of MicroRNAs in Diabetic Nephropathy” by H. Wu et al. provides an overview of the potential involvement of these small noncoding RNAs in DN. This comprehensive review leads to a more thorough understanding of the pathogenesis of DN at the molecular level. It may be hoped that some of these molecules may, in the foreseeable future, serve as potential targets for pharmaceutical intervention to prevent or at least delay DN.

In their review article entitled “Histone Lysine Methylation in Diabetic Nephropathy,” G. Sun et al. discuss in detail the role of histone methylation in the pathogenesis and progression of DN and summarize recent advances in the field of epigenetic modifications of genes. All in all, histone methylation and other epigenetic modifications of DNA open up a new vista in our understanding of how DN can develop under certain interference between genes and environment. 

*(d) Diabetic Retinopathy*. Nowadays, the importance of adequate glycemic control in the prevention of diabetic retinopathy (DR) is well established [8]. However, other factors may play a role in the pathogenesis of this complication of diabetes. There are an increasing number of studies on the associations of DR with various polymorphisms of genes such as vascular endothelial growth factor (VEGF) and eNOS gene [9, 10].

L. Han et al. in their paper titled “The Associations between VEGF Gene Polymorphisms and Diabetic Retinopathy Susceptibility: A Meta-Analysis of 11 Case-Control Studies” performed a comprehensive meta-analysis to evaluate the relationship between 5 VEGF gene polymorphisms and susceptibility to DR. Significant associations were found between DR susceptibility and two out of five VEGF gene polymorphisms. These findings add to the growing body of literature on the role of VEGF polymorphisms in DR risk.

Another meta-analysis by Z. Ma et al. in the paper entitled “Association between eNOS 4b/a Polymorphism and the Risk of Diabetic Retinopathy in Type 2 Diabetes Mellitus: A Meta-Analysis” showed a lack of association between eNOS-4b/a polymorphism and the risk of DR. Although this meta-analysis was based mainly on Asian populations, it provides a better understanding of the relationship between eNOS-4b/a gene polymorphisms and DR.

Y. Lin et al. in their paper entitled “Serum Fibroblast Growth Factor 21 Levels Are Correlated with the Severity of Diabetic Retinopathy” found higher fibroblast growth factor 21 (FGF21) concentration in patients with diabetic retinopathy. These findings are used as evidence for a potential role of FGF21 in the pathogenesis of diabetic retinopathy. However, larger prospective studies are needed to establish this relationship.

Focusing on T1DM, S. B. Polat et al. in their article entitled “Evaluation of Serum Fibrinogen, Plasminogen, *α*2-Anti-Plasmin, and Plasminogen Activator Inhibitor Levels (PAI) and Their Correlation with Presence of Retinopathy in Patients with Type 1 DM” compared serum fibrinogen, plasminogen, *α*2-anti-plasmin, and PAI levels in T1DM patients with different degrees of DR and in healthy voluntaries. Subjects with T1DM had significantly higher *α*2-anti-plasmin levels, and these levels exhibit a positive correlation with HbA_1c_. Clearly, this study adds new useful information and leads towards a better understanding of the fibrinolytic and thrombotic status in the pathogenesis of DR in T1DM.

In their paper entitled “Vitamin Status as a Determinant of Serum Homocysteine Concentration in Type 2 Diabetic Retinopathy,” P. Fotiou et al. found that patients with DR exhibited higher serum homocysteine and lower vitamin B_12_ and serum folic acid levels, as compared with those who did not have DR. Based on their findings, the authors claimed that hyperhomocysteinemia, especially when accompanied by low folic acid and vitamin B_12_ levels, can serve as an independent risk factor for DR.

A. Praidou et al. in the paper entitled “Diabetic Retinopathy Treated with Laser Photocoagulation and the Indirect Effect on Glycaemic Control” reported that the patients with DR who had been treated with laser photocoagulation showed better glycemic control compared with untreated patients with DR. These unexpected findings suggest that treatment of DR with laser photocoagulation might have a psychological effect on patients, motivating them to improve their lifestyle and their adherence to treatment.

Z. Torok et al. in their paper entitled “Combined Methods for Diabetic Retinopathy Screening, Using Retina Photographs and Tear Fluid Proteomics Biomarkers” aimed to develop a novel automated method for DR screening. The authors ended up with a combined screening method for detection of microaneurysms, which resulted in a significant improvement of both sensitivity and specificity. Taken together, these findings raise hope for a more promising and accurate diagnostic modality for DR. 

*(e) Macrovascular Complications*. Prior studies have proposed that measuring biomarkers of atherosclerosis can be helpful in detecting macrovascular complications of diabetes, such as subclinical carotid disease [11].

The clinical study described in the paper entitled “Relationship between HgbA1c and Myocardial Blood Flow Reserve in Patients with Type 2 Diabetes Mellitus: Noninvasive Assessment Using Real-Time Myocardial Perfusion Echocardiography” by R. Huang et al. aimed to scrutinize the relationship between HbA_1c_ and myocardial perfusion in patients with T2DM. The study showed that optimal glycemic control is associated with a preservation of myocardial blood flow reserve (MBFR) in T2DM patients who are at risk for CAD. Undeniably, this study provides robust clinical evidence that macrovascular complications of diabetes can be prevented by early and tight glycemic control.

The article entitled “Cumulative Effects of Hypertension, Dyslipidemia, and Chronic Kidney Disease on Carotid Atherosclerosis in Chinese Patients with Type 2 Diabetes Mellitus” by C. Yuan et al. showed that patients with multiple risk factors for atherosclerosis exhibit higher carotid plaque score, as compared with those having fewer risk factors. This work adds to the growing body of literature on the utility of risk factors estimated by ultrasound for the diagnosis and prognosis of carotid atherosclerosis in T2DM patients.

M. Celik et al. in their work entitled “Relation of Asymmetric Dimethylarginine Levels to Macrovascular Disease and Inflammation Markers in Type 2 Diabetic Patients” reported that serum asymmetric dimethylarginine (ADMA) levels were significantly higher in patients with T2DM and macrovascular complications. These findings are important because they highlight the potential role of ADMA in the pathogenesis of atherosclerosis in T2DM.

J.-M. Chen et al. in their work titled “Acarbose Treatment and the Risk of Cardiovascular Disease in Type 2 Diabetic Patients: A Nationwide Seven-Year Follow-Up Study” used nationwide insurance claim dataset to scrutinize if there is a benefit of acarbose treatment on cardiovascular disease (CVD) in T2DM. The authors found that patients receiving acarbose showed a lower risk of developing CVD, despite the fact that they exhibited a higher hazard ratio for CVD during the first 12 months of this treatment. These findings ensure a more thorough view of the protective role of acarbose in the cardiovascular system. 

*(f) Miscellaneous Complications*. Diabetic foot is a major complication of diabetes characterized by the presence of chronic ulcers that often fail to heal. In the pathogenesis of chronic ulcers, matrix metalloproteinases may play a pivotal role [12]. L. Li et al. in their study entitled “The Effect of Autologous Platelet-Rich Gel on the Dynamic Changes of the Matrix Metalloproteinase-2 and Tissue Inhibitor of Metalloproteinase-2 Expression in the Diabetic Chronic Refractory Cutaneous Ulcers” examined the effect of autologous platelet-rich gel (APG), used as treatment of diabetic chronic refractory cutaneous ulcers, in the expression of matrix metalloproteinases (MMPs), tissue inhibitor of metalloproteinases (TIMPs), and transforming growth factor- (TGF-) *β*1. This study enriches our knowledge on the beneficial effect of APG on recalcitrant diabetic cutaneous wound healing.

Statins are commonly used drugs that may have negative effect on glycemic control in patients with diabetes [13]. J. Zhou et al. in their article entitled “Effects of Simvastatin on Glucose Metabolism in Mouse MIN6 Cells” investigated the effects of simvastatin on insulin secretion in mouse MIN6 cells. Based on their findings, the authors proposed that simvastatin might inhibit insulin synthesis and secretion, thereby impairing glucose metabolism. These results are of paramount importance, given the widespread use of statins as drugs preventing cardiovascular disease.

Diabetes Self-Management Education (DSME) interventions have proven to help T2DM patients achieve and maintain optimal glycemic control [14]. The aim of the study “The Effect of Diabetes Self-Management Education on Body Weight, Glycemic Control, and Other Metabolic Markers in Patients with Type 2 Diabetes Mellitus” by C. Yuan et al. was to evaluate the effect of a short-term DSME on metabolic markers and atherosclerotic parameters in patients with T2DM. This work adds to the growing body of literature on the benefits that DSME can provide in diabetic patients.

Postprandial dyslipidemia is a common disorder in diabetes and it has been recognized as an important risk factor for CVD [15]. In their work entitled “Study of Postprandial Lipaemia in Type 2 Diabetes Mellitus: Exenatide versus Liraglutide,” M. Voukali et al. investigated the effect of two GLP-1 receptor agonists, exenatide and liraglutide, on postprandial dyslipidemia. Both GLP-1 receptor agonists were equally effective in reducing postprandial lipemia. These findings give a more complete picture on the multiple mechanisms of action of GLP-1 receptor agonists.

Epidemiologic studies have shown an increased cancer incidence in diabetic patients, but the underlying mechanisms remain obscure [16]. The paper entitled “Real Life Cancer Comorbidity in Greek Patients with Diabetes Mellitus Followed Up at a Single Diabetes Center: An Unappreciated New Diabetes Complication” by A. Thanopoulou et al. aimed to determine cancer comorbidity in Greek patients with both diabetes types and to investigate the related risk factors. The authors suggest that we should develop strategies for early detection and prevention of certain types of cancer in diabetes. 

*Conclusions*. All research and review papers included in this special issue highlight the fact that enquiry into diabetic complications is progressing. Although the papers published in the issue represent a small sample of the research in the field, they underline the complexity of the various etiological mechanisms and they illustrate the need of a comprehensive approach for the early detection and efficacious management of these chronic complications. Thus, we hope that the readers will find the content in this special issue to be a valuable resource for a better understanding of these important issues. This progress made in the field of diabetes research notwithstanding, we still have a long way to go until we succeed in unraveling the pathogenic mechanisms underlying these complications and in finding definitive cures.
